# Knowledge of HBV and HCV and Individuals’ Attitudes Toward HBV- and HCV-Infected Colleagues: A National Cross-Sectional Study among a Working Population in Japan

**DOI:** 10.1371/journal.pone.0076921

**Published:** 2013-09-26

**Authors:** Hisashi Eguchi, Koji Wada

**Affiliations:** 1 Environment and Safety Division, Kyocera Corporation Shiga–Gamou Plant, Higashiomi, Shiga, Japan; 2 National Institute for Global Health and Medicine, Shinjuku, Tokyo, Japan; Saint Louis University, United States of America

## Abstract

Prejudice and discrimination in the workplace regarding the risk of transmission of Hepatitis B virus (HBV) and Hepatitis C virus (HCV) are increased by excess concerns due to a lack of relevant knowledge. Education to increase knowledge about HBV and HCV and their prevention could be the first step to reduce prejudice and discrimination. This study aimed to determine the association between the level of knowledge and negative attitudes toward HBV- and HCV-infected colleagues among the Japanese working population. An online anonymous nationwide survey involving about 3,000 individuals was conducted in Japan. The questionnaire consisted of knowledge of HBV and HCV, and attitudes toward HBV- and HCV-infected colleagues in the workplace. Knowledge was divided into three categories: “ensuring daily activities not to be infected”; “risk of infection”; and “characteristics of HBV/HCV hepatitis”, based on the result of factor analysis. Multiple logistic regression analysis was applied. A total of 3,129 persons responded to the survey: 36.0% reported they worried about the possibility of transmission of HBV and HCV from infected colleagues; 32.1% avoided contact with infected colleagues; and 23.7% had prejudiced opinions about HBV and HCV infection. The participants were classified into tertiles. A higher level of knowledge of HBV and HCV was significantly associated with these three negative attitudes (*P* for trend < 0.005). This study suggests that increasing knowledge may decrease individuals’ negative attitudes towards HBV- and HCV-infected colleagues. Thus, we should promote increased knowledge of HBV and HCV in stages to reduce negative attitudes toward HBV- and HCV-infected colleagues.

## Introduction

Although the risk of transmission of HBV and HCV through daily contact in the workplace is very low, many patients perceive prejudice and discrimination from acquaintances, family members, and even health care providers [1,2]. Fear of the possibility of transmission, where the perceived risk is often unnecessarily inflated, could also result in unnecessary changes in everyday practices [3]. For patients with HCV, the greatest concern was transmitting the virus to family members [4]. Patients with emerging infectious diseases such as HIV and SARS face problems obtaining a job and perceive unfair treatment in the workplace [5,6]. In Japan, prejudice and discrimination in the workplace also result from the idea that infection with HBV and HCV is misunderstood to be similar to that of HIV [7].

Education to increase public knowledge of a specific disease is often the first step to reduce prejudice and discrimination [8,9]. Targeting inaccurate beliefs about viral hepatitis might improve public health interventions to foster healthier behavior and better hepatitis outcomes [10]. Increasing knowledge of HBV and HCV is effective for preventing the acquisition and spread of infection [11-13]. However, reducing the stigma about HIV/AIDS and mental illness has not always yielded better outcomes [14], [15]. Educational efforts were effective in improving knowledge of HIV/AIDS transmission but these efforts did not convince the general public that HIV/AIDS could not be transmitted through casual contact [16].

It is reasonable to suggest that increasing knowledge of HBV and HCV might reduce negative attitudes towards HBV- and HCV-infected colleagues in the workplace. As far as we are aware, no studies have explored the association between knowledge of HBV and HCV and individuals’ attitudes toward HBV- and HCV-infected colleagues in the workplace. Therefore, the aim of this study was to determine the association between higher levels of knowledge and negative attitudes toward HBV- and HCV-infected colleagues among the Japanese working population.

## Materials and Methods

### Participants and conduct of the survey

An online, anonymous, self-administered questionnaire was sent to 7,937 individuals in 47 prefectures of 10 areas in Japan in October 2011. Participants were selected randomly from volunteers registered with a survey company, using a stratified sampling method with sex and age. The sex ratio was 1:1, and there were equal numbers of participants in each group.

The cross-sectional survey comprised 28 questions ranging from participants’ demographics (five items), to knowledge of essential factors concerning HBV and HCV (19 items), and general attitudes toward HBV- and HCV-infected colleagues (three items), accompanied by one question related to physical condition. Participants’ demographics information comprised sex, age, educational level, occupation, and individual income.

### Demographics and basic characteristics of participants

The demographics and basic characteristics of participants are shown in Table 1. Age was classified into five groups: 20–29, 30–39, 40–49, 50–59 and 60–69 years. Educational level was divided into three categories of lower than or equal to high school graduation; technical college or junior college; and higher than university. Occupation was classified into five groups: regular employee, non-regular employee, unemployed, others, and undergraduates. Others included agriculture, fishery, forestry, and self-employed business owners. Individual income was classified in three equal groups: low, <1 million yen (<12,500 US$); middle, 1–3 million yen (12,500–37,500 US$); and high, >3 million yen (>37,500 US$) (1 US$=80 yen). “Physical condition” was assessed by asking the following question, “How about your health status?” Responses were measured on a four-point scale (1 = very healthy; 2 = relatively healthy; 3 = relatively unhealthy; and 4 = unhealthy), and they were further dichotomized into “very healthy” and “relatively healthy” (= 1) and others (= 0).

**Table 1:** Demographics and basic characteristics of participants (n=3,129).

		n	(%)
**Gender**			
	Male	1,549	(49.5)
	Female	1,580	(50.5)
**Age (yr)**			
	20-29	618	(19.8)
	30-39	628	(20.1)
	40-49	627	(20.0)
	50-59	632	(20.2)
	60-69	624	(19.9)
**Education**			
	Junior high school or high school	693	(22.1)
	Technical college or junior college	572	(18.3)
	University and graduate school	1,864	(59.6)
**Occupation**			
	Regular employee	1,076	(34.4)
	Non-regular employee	540	(17.3)
	Unemployed	1,010	(32.3)
	Others	292	(9.3)
	Undergraduate student	211	(6.7)
**Healthy status**			
	Relatively healthy	2,627	(84.0)
	Others	502	(16.0)
**Individual income**			
	Low (<1 million yen/year)	1,236	(39.0)
	Middle (1-3 million yen/year)	842	(26.6)
	High (>3 million yen/year)	1,091	(34.4)

**Attitude towards HBV/HCV infected colleagues**			
	**Worrying about transmission**		
	Strongly agree and agree	1,125	(36.0)
	Disagree and strongly disagree	2,004	(64.0)
	**Avoiding contact with infected colleagues**		
	Strongly agree and agree	1,003	(32.1)
	Disagree and strongly disagree	2,126	(67.9)
	**Having prejudiced opinions about infected colleagues**		
	Strongly agree and agree	742	(23.7)
	Disagree and strongly disagree	2,387	(76.3)

### Knowledge of HBV and HCV, and general attitudes toward HBV- and HCV-infected colleagues

Knowledge of HBV and HCV was investigated with 19 items that were developed from a discussion based on previous studies [8] [17,18]. Responses were measured on a two-point scale (0= No, I did not know, and 1= Yes, I knew). Table 2 shows each question. Cronbach α was calculated for each factor. We summed each factor, with the greater scores being indicative of having knowledge related to HBV or HCV. The participants were classified into tertiles (low, middle and high) according to the score for each category.

**Table 2:** Association of each negative attitude (n=3,129).

Worrying about transmission	Avoiding contact with infected colleagues	Having prejudiced opinions about infected colleagues	n	(%)
Strongly agree and agree	Strongly agree and agree	Strongly agree and agree	534	(17.1)
		Disagree and strongly disagree	336	(10.7)
	Disagree and strongly disagree	Strongly agree and agree	38	(1.2)
		Disagree and strongly disagree	217	(6.9)
Disagree and strongly disagree	Strongly agree and agree	Strongly agree and agree	52	(1.7)
		Disagree and strongly disagree	81	(2.6)
	Disagree and strongly disagree	Strongly agree and agree	118	(3.8)
		Disagree and strongly disagree	1753	(56.0)

We used the following attitudes toward HBV and HCV infection as the outcome variables of the study: (1) “If I found that people with whom I work were infected with hepatitis virus, I would become anxious that I may be infected too” (

“worrying about transmission”); (2) “If I found that people with whom I work were infected with hepatitis virus, I think I would try not to come in contact with him/her as much as possible” (“avoiding contact with infected colleagues”); and (3) “If I found that people with whom I work were infected with hepatitis virus, I think I would look at him/her erroneously with prejudice, suspecting him/her to be a homosexual, someone who engaged in sexual relationships with an unspecified number of people, or a drug addict” (“Having prejudiced opinions about infected colleagues”). Responses for all three outcomes were measured on a four-point scale (1= I think so; 2= I think so to a degree; 3= I do not really think so; and 4= I do not think so at all), and they were further dichotomized into “I think so” and “I think so to a degree” (=1) and the others (= 0).

### Statistical analysis

We assessed factor structures of the three constructs using exploratory factor analysis, whereby all 19 items were entered using the maximum likelihood method. Factors were extracted using common-factors analysis, followed by promax rotation. We named each factor originally based on our discussion.

To explore associations between participants’ knowledge of HBV and HCV and their attitudes toward HBV- and HCV-infected colleagues, each statement of opinion about infected colleagues was treated as a separated outcome. Multiple logistic regression analysis was used to determine the association between level of knowledge of HBV/HCV and the three negative attitudes towards HBV/HCV infected colleagues, where factors included in multivariate analysis were sex, age, educational level, occupation, individual income, and health status. With respect to knowledge of HBV and HCV, the score for each domain was entered into the model. The outcome was not rare; therefore, we applied Zhang’s formula [19].

Statistical analysis was performed using SPSS version 17.0 (IBM, Chicago, IL, USA). A two-tailed value of *P* < 0.05 was considered significant unless otherwise indicated.

### Ethics

This survey was approved by the institutional ethics committee of Kitasato University School of Medicine. Response to the questionnaire was taken as agreement to participate in the study.

## Results

### Participant demographics and negative attitudes toward HBV and HCV infection

Our recruitment was terminated when the number of participants reached 3,129. Of the 3,129 respondents, 1,549 (49.5%) were male and each age group (20–29, 30–39, 40–49, 50–59, and 60–69 years old) contained approximately 20% of the total number of participants. The sample included 32.3% unemployed persons and 6.7% undergraduate students. Table 1 summarizes the characteristics of the study participants.

In terms of participants’ negative attitudes towards HBV and HCV infection, 1,125 (36.0%) would worry about the possibility of transmission if they worked together with an infected colleague at the same workplace (worrying about transmission); 1,003 (32.1%) would avoid contact with HBV- and HCV-infected colleagues (avoiding contact with infected colleagues); and 742 (23.7%) would have a prejudiced opinion about their HBV- and HCV-infected colleagues (having prejudiced opinions about infected colleagues) (Table 1). “Disagree and strongly disagree” of all three negative attitudes was 56.0%; “Strongly agree and agree” of all three negative attitudes was 17.1% (Table 2).

### Individuals’ levels of knowledge about HBV and HCV

Table 3 shows the results of exploratory factor analyses. Three factors were extracted. Factor 1 consisted of six items regarding “ensuring daily activities not to be infected”; Factor 2 consisted of five items regarding “risk of infection”; and finally, Factor 3 consisted of eight items regarding “characteristics of hepatitis”. Cronbach α coefficient was 0.93 for “ensuring daily activities not to be infected”, 0.88 for “risk of infection”, and 0.93 for “characteristics of viral hepatitis”.

**Table 3:** Exploratory factor analysis of 19 items of the questions about hepatitis virus using the maximum likelihood method and Promac rotation (n=3,124).

Items:	Answer (%)		Factor 1	Factor 2	Factor 3	Category name	Cronbach’s α
	Yes	No					
HBV/HCV not transmitted even if you share the same tableware with someone who is infected	60.4	29.6	-0.126	0.057	0.910	Ensuring daily activities not to be infected	0.93
HBV/HCV not transmitted even if you lightly kiss someone who is infected	45.2	54.8	-0.217	0.167	0.790		
HBV/HCV not transmitted even if you use the same bath, such as a hot spring, with someone who is infected	62.1	37.9	0.017	0.091	0.769		
HBV/HCV not transmitted even if you talk with someone who is infected	76.5	23.5	0.385	-0.111	0.642		
HBV/HCV not transmitted even if you work with someone who is infected	76.6	23.4	0.412	-0.108	0.624		
HBV/HCV not transmitted even if you shake hands with someone who is infected	76.7	23.3	0.411	-0.113	0.606		

HBV/HCV spread via the blood and body fluids of infected persons	77.8	22.2	0.918	-0.007	-0.097	Risk of infection	0.88
Blood tests can tell you if you are infected with HBV/HCV	77.6	22.4	0.812	0.036	-0.026		
Those who have received a blood transfusion or blood preparations in the past may be infected with HBV/HCV	78.4	21.6	0.784	0.068	-0.075		
HBV/HCV may be transmitted if you share a razor, pierced earrings, a syringe, etc., with someone who is infected	71.6	28.4	0.737	-0.001	0.026		
HBV/HCV may be transmitted if you have sexual intercourse with someone who is infected.	63.7	36.3	0.628	0.048	0.025		

HBV/HCV is more often the cause of hepatic cirrhosis than drinking alcohol; it accounts for roughly 85% of cases	21.3	78.7	-0.106	0.777	0.050	Characteristics of HBV/HCV hepatitis	0.86
HBV/HCV is the cause of liver cancer in approximately 90% of cases	18.5	81.5	-0.141	0.760	0.062		
Some people who are persistently infected with HBV/HCV may develop hepatic cirrhosis or liver cancer at age 40–60 years	38.7	61.3	0.125	0.631	0.044		
Even though the results of a liver function test produced no abnormal results, you may be persistently infected with HBV/HCV	29.0	71.0	0.076	0.630	0.009		
By having hepatitis B or C adequately treated, you may be able to completely cure it, or delay the advancement of cirrhosis or liver cancer	39.1	60.9	0.154	0.625	0.021		
1 in 50 Japanese people aged 15–59 years are assumed to be persistently infected with HBV/HCV	12.0	88.0	-0.110	0.584	0.051		
Even though you may not have any special subjective symptoms, you may be persistently infected with HBV/HCV	46.9	53.1	0.283	0.534	-0.033		
Patients infected with hepatitis virus may need to discontinue work to undergo treatment	42.8	57.2	0.267	0.459	-0.065		
Variance explained (%)			42.9	10.8	4.8		

By univariate analysis, a high level knowledge of HBV and HCV for each category was significantly associated with worrying about transmission, avoiding contact with infected colleagues, and having a prejudiced opinion about infected colleagues. These associations remained after adjusting for potential confounders by multivariate analysis (Tables 4 and 5). Worrying about transmission was associated with knowledge of HBV and HCV: moderate level [adjusted odds ratio (OR) “ensuring daily activities not to be infected”, “risk of infection”, and “characteristics of viral hepatitis”: 0.74, 0.91, 0.92; 95% confidence interval (CI): 0.66–0.81, 0.83–1.00, and 0.85–0.99; and high level (OR: 0.60, 0.82, and 0.80; 95%CI: 0.54–0.66, 0.75–0.89, and 0.73–0.86). Avoiding contact with infected colleagues was associated with knowledge of HBV and HCV: moderate level (OR: 0.76, 0.76, and 0.88; 95% CI: 0.69–0.83, 0.68–0.83, and 0.81–0.94); and high level (OR: 0.64, 0.54, and 0.79; 95% CI: 0.58–0.70, 0.48–0.60, and 0.73–0.85). Having prejudiced opinions about infected colleagues was associated with knowledge of HBV and HCV: moderate level (OR: 0.90, 0.95, and 0.93; 95% CI: 0.84–0.96, 0.89–1.01, and 0.88–0.98); and high level (OR: 0.87, 0.92, and 0.89; 95% CI: 0.81–0.91, 0.87–0.97, and 0.83–0.94). A higher level of knowledge of HBV and HCV was significantly associated with these three negative attitudes (*P* for trend < 0.005).

**Table 4:** Demographics of each negative attitude and each knowledge about HBV and HCV.

		n=3,129	(%^a^)	Worrying about transmission		Avoiding contact infected colleagues		Having prejudiced opinions about infected colleagues	
				n=1,125	(%^b^)	n=1,003	(%^b^)	n=742	(%^b^)
Ensuring daily activities not to be infected									
Low	(0-3)	685	(21.9)	311	(45.4)	279	(40.7)	198	(28.9)
Moderate	(4-5)	764	(24.4)	294	(38.5)	253	(33.1)	183	(24.0)
High	(6)	1680	(53.7)	520	(31.0)	471	(28.0)	361	(21.5)

Risk of infection									
Low	(0-2)	1,083	(34.6)	475	(43.9)	441	(40.7)	314	(29.0)
Moderate	(3-4)	1,038	(33.2)	388	(37.4)	327	(31.5)	240	(23.1)
High	(5)	1,008	(32.2)	262	(26.0)	235	(23.3)	188	(18.7)

Characteristics of HBV/HCV hepatitis									
Low	(0)	1,027	(32.8)	555	(54.0)	502	(48.9)	318	(31.0)
Moderate	(1-3)	747	(23.9)	273	(36.5)	237	(31.7)	163	(21.8)
High	(4-8)	1,355	(43.3)	297	(21.9)	264	(19.5)	261	(19.3)

a The percentage indicates the number of each tertiles (low, moderate, high) divided by total number (3,129)

b The percentage indicates the number of "yes" answers divided by total number of each tertiles (low, moderate, high)

HBV, Hepatitis B virus; HCV, Hepatitis C virus.

**Table 5:** Univariate and multivariate analyses of association between each domain of HBV/HCV knowledge and attitudes toward HBV/HCV infection (n=3,129).

		Odds ratio (95% confidence interval)					
		Worrying about transmission		Avoiding contact with infected colleagues		Having prejudiced opinions about infected colleagues	
		Univariate model	Multivariate model^ab^	Univariate model	Multivariate model^ab^	Univariate model	Multivariate model^ab^
Ensuring daily activities not to be infected							
Low^c^	(0–3)	ref	ref	ref	ref	ref	ref
Moderate	(4–5)	0.72 (0.65–0.80)	0.74 (0.66–0.81)	0.75 (0.68–0.82)	0.76 (0.69–0.83)	0.88 (0.82–0.94)	0.90 (0.84–0.96)
High	(6)	0.59 (0.53–0.65)	0.60 (0.54–0.66)	0.63 (0.58–0.69)	0.64 (0.58–0.70)	0.85 (0.80–0.90)	0.87 (0.81–0.91)
Test for linear trend^d^		p<0.001	p<0.001	p<0.001	p<0.001	p<0.001	p<0.001

Risk of infection							
Low^c^	(0–2)	ref	ref	ref	ref	ref	ref
Moderate	(3–4)	0.89 (0.80–0.97)	0.91 (0.83–1.00)	0.89 (0.81–0.96)	0.76 (0.68–0.83)	0.93 (0.87–0.99)	0.95 (0.89–1.01)
High	(5)	0.79 (0.72–0.86)	0.82 (0.75–0.89)	0.82 (0.76–0.88)	0.54 (0.48–0.60)	0.91 (0.85–0.96)	0.92 (0.87–0.97)
Test for linear trend^d^		p<0.001	p<0.001	p<0.001	p<0.001	p<0.001	p=0.002

Characteristics of HBV/HCV hepatitis							
Low^c^	(0)	ref	ref	ref	ref	ref	ref
Moderate	(1-3)	0.72 (0.65–0.80)	0.92 (0.85–0.99)	0.87 (0.80–0.93)	0.88 (0.81–0.94)	0.92 (0.87–0.97)	0.93 (0.88–0.98)
High	(4-8)	0.59 (0.53–0.65)	0.80 (0.73–0.86)	0.77 (0.71–0.83)	0.79 (0.73–0.85)	0.87 (0.82–0.92)	0.89 (0.83–0.94)
Test for linear trend^d^		p<0.001	p<0.001	p<0.001	p<0.001	p<0.001	p<0.001

a Each domain was entered into multivariate model separately

b Adjusted for sex, age, education, occupation, healthy status, individual income

c Reference category

d Test for linear trends were performed by modeling the group scores of HBV/HCV knowledge (1, 2, 3) as one variable.

## Discussion

This study aimed to evaluate the association between level of knowledge of HBV and HCV and individuals’ negative attitudes toward HBV- and HCV-infected colleagues in the workplace in Japan. We hypothesized that a high level of knowledge of HBV and HCV would be associated with decreasing negative attitudes toward HBV- and HCV-infected colleagues. Our study showed that increasing knowledge of HBV and HCV in each of three categories was associated with decreasing negative attitudes towards HBV- and HCV-infected colleagues.

The level of HBV/HCV knowledge in the Japanese working population was acceptable and in the present study, it was almost the same as in a previous studies of Australian and Chinese health professionals [2] and higher than in the general population [10-12,17,18]. This result indicated that Japanese working population had a relatively high knowledge of HBV/HCV hepatitis.

Knowledge of HBV/HCV was divided into three categories: “Ensuring daily activities not to be infected”, “Risk of infection” and “Characteristics of HBV/HCV hepatitis”. The level of knowledge of “Characteristics of HBV/HCV hepatitis” was lower than the level of knowledge about “Risk of infection” and “Ensuring daily activities not to be infected”. Knowledge of “Characteristics of HBV/HCV hepatitis”, especially “HBV/HCV is the cause of liver cancer in approximately 90% of cases” and “by having hepatitis B or C adequately treated, you may be able to completely cure it, or delay the advancement of cirrhosis or liver” was relatively low. This result is consistent with previous studies [10,11]. The level of knowledge regarding treatment and prognosis of HIV/AIDS was higher than the level of knowledge of HCV [10]. Education to improve knowledge regarding infectious transmission and preventive measures as well as characteristics of hepatitis such as prognosis and treatment should be implemented.

Although HBV/HCV cannot be transmitted through casual contact in the workplace, 20% to 40% of participants had a negative attitude toward HBV/HCV-infected colleagues, 56% of participants did not have all three negative attitudes, and 17% of participants had all three negative attitudes. Negative attitudes towards HBV and HCV infection were evaluated by three items such as “worrying about transmission” (awareness), “avoiding contact with infected colleagues” (behavior) and “having prejudiced opinions about infected colleagues” (discrimination). The percentages of each item were “awareness” (36.0%), “behavior” (32.1%) and “discrimination” (23.7%). In a previous study of HIV/AIDS, “awareness” was more difficult to improve by education compared with other negative attitudes [8]. This may be influenced by inflating the risk of transmission [3]. It might suggest that decreasing negative attitude may be in order of "discrimination", "behavior", and "awareness".

Increasing the level of knowledge regarding HBV/HCV was associated with decreasing negative attitudes towards HBV/HCV-infected colleagues in the workplace. Community-based studies suggest that increasing the level of knowledge of HIV/AIDS and tuberculosis by education leads to a decrease in negative attitudes towards infected patients [15,20,21]. However, even health care professionals with high levels of knowledge regarding HBV/HCV showed discrimination towards hepatitis patients. People living with HIV/AIDS are subjected to stigma, which is significantly associated with organizational cynicism [22]. Thus, both education that provident knowledge and problem solving, learning and interactive educational sessions are recommended [2]. A multidimensional educational approach to increase the awareness of HBV/HCV may be needed in the workplace.

The strength of the present study was that it involved a large sample of more than 3,000 participants from all regions of Japan. Furthermore, the participants had different professions and included homemakers, who are common in Japan, which enabled a wide generalization of the findings. There were some limitations of the study. Our study population presumably had internet access and therefore might have been more aware of HBV and HCV through access to online information [23]. Our study population was educated to a higher level (60% of subjects were university and graduate school) than the general Japanese working population. The study was cross-sectional; therefore, no causal relationship could be concluded from the findings. To clarify the causal relationship between knowledge of HBV/HCV and negative attitudes, an interventional study should be conducted in the future. Although HBV and HCV have different disease characteristics with different dominant modes of transmission and different types and goals of therapy, we did not measure knowledge of HBV and HCV separately. Furthermore, although knowledge of HBV and HCV were probable contributors to attitudes towards HBV- and HCV-infected colleagues, factors influencing their level of knowledge remain unknown. In addition, only some indicators of knowledge regarding HBV and HCV and attitudes towards HBV- and HCV-infected colleagues were investigated.

## Conclusion

This study suggests that increasing knowledge may improve individuals’ negative attitudes towards HBV- and HCV-infected colleagues. We should promote increased knowledge of HBV and HCV in stages to reduce negative attitudes towards HBV- and HCV-infected colleagues.
